# Supplementary figures and images for: What is the nature of motor adaptation to dynamic perturbations?
Source: PLoS Comput Biol. 2022 Aug 30;18(8):e1010470. doi: 10.1371/journal.pcbi.1010470 (PMC9467354; doi:10.1371/journal.pcbi.1010470)

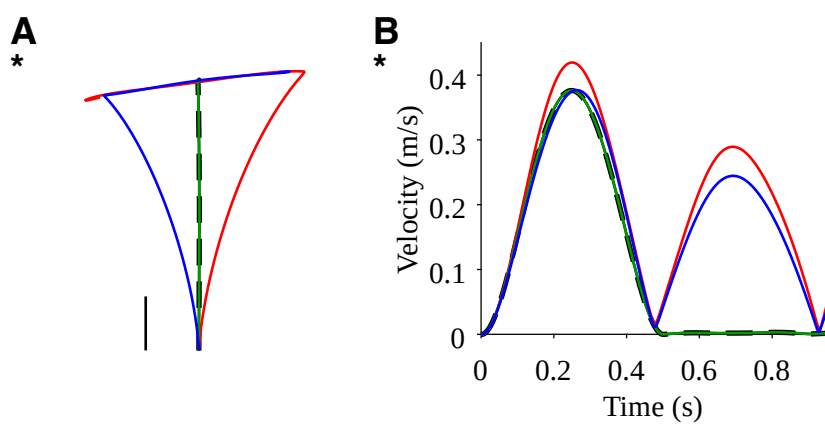

Figure S1

Supplement: S1 Fig — A. Simulated trajectories. B. Simulated velocity profiles. (PDF) [file pcbi.1010470.s001.pdf]

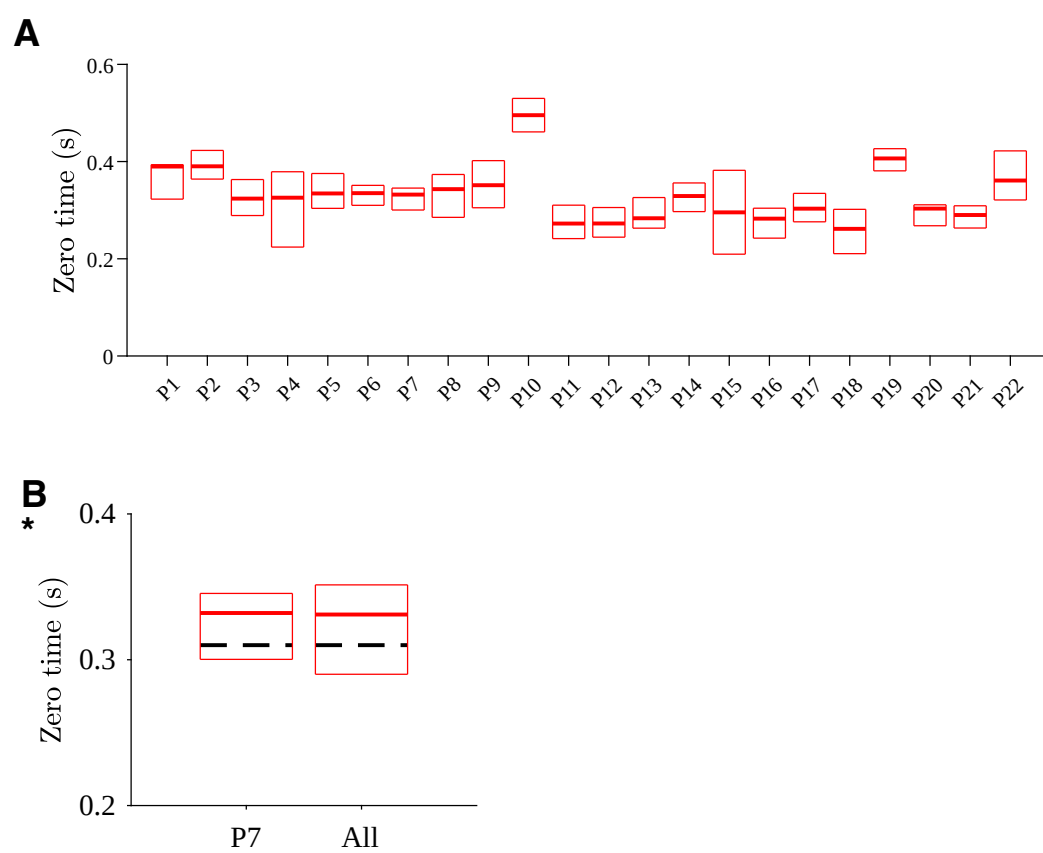

Figure S2

Supplement: S2 Fig — A. All participants with mean value (thick line) and 25–75 percentiles (box). B. Data of participant P7 and mean of all the participants. The black dashed line is the model prediction. (PDF) [file pcbi.1010470.s002.pdf]

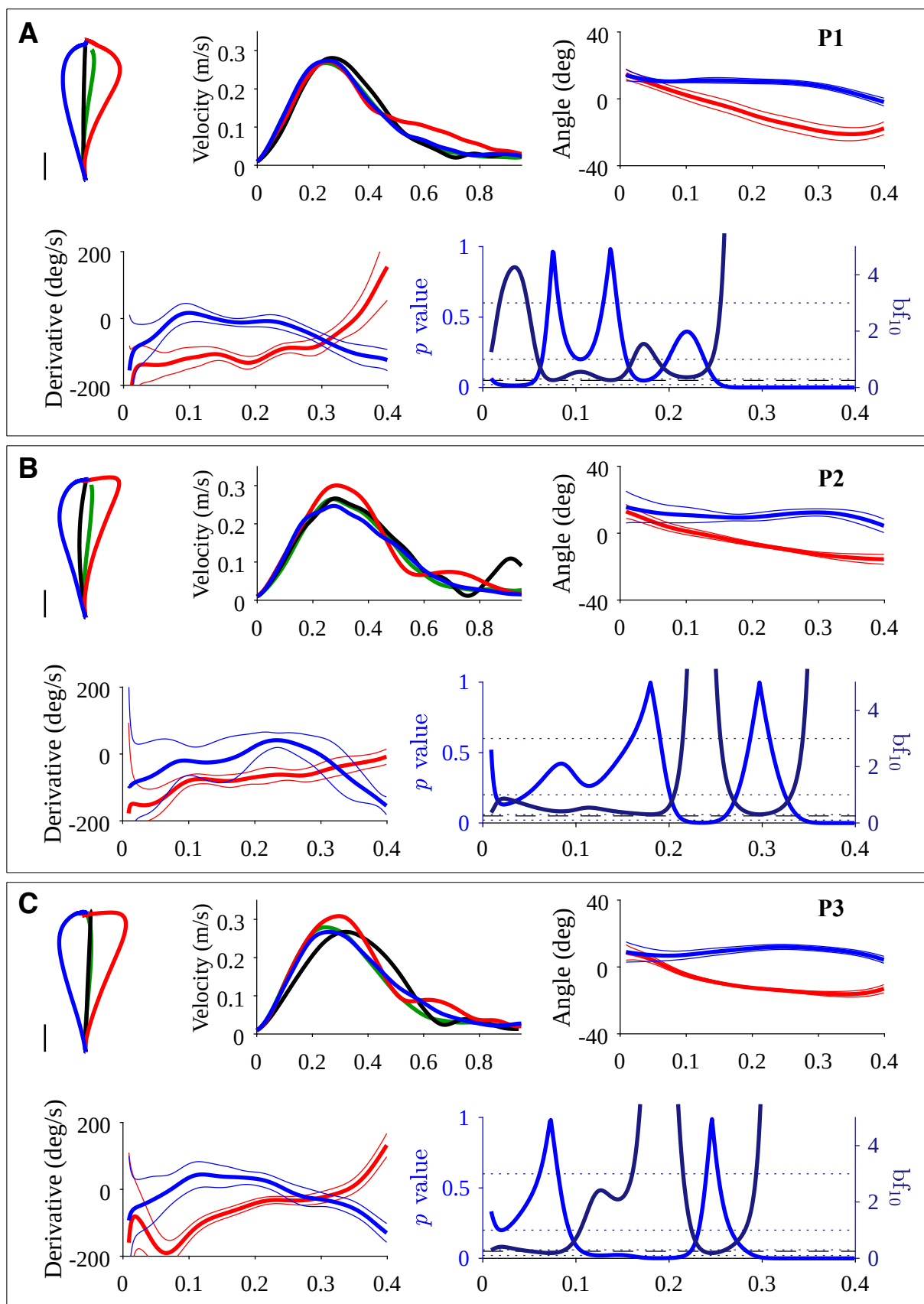

Figure S3

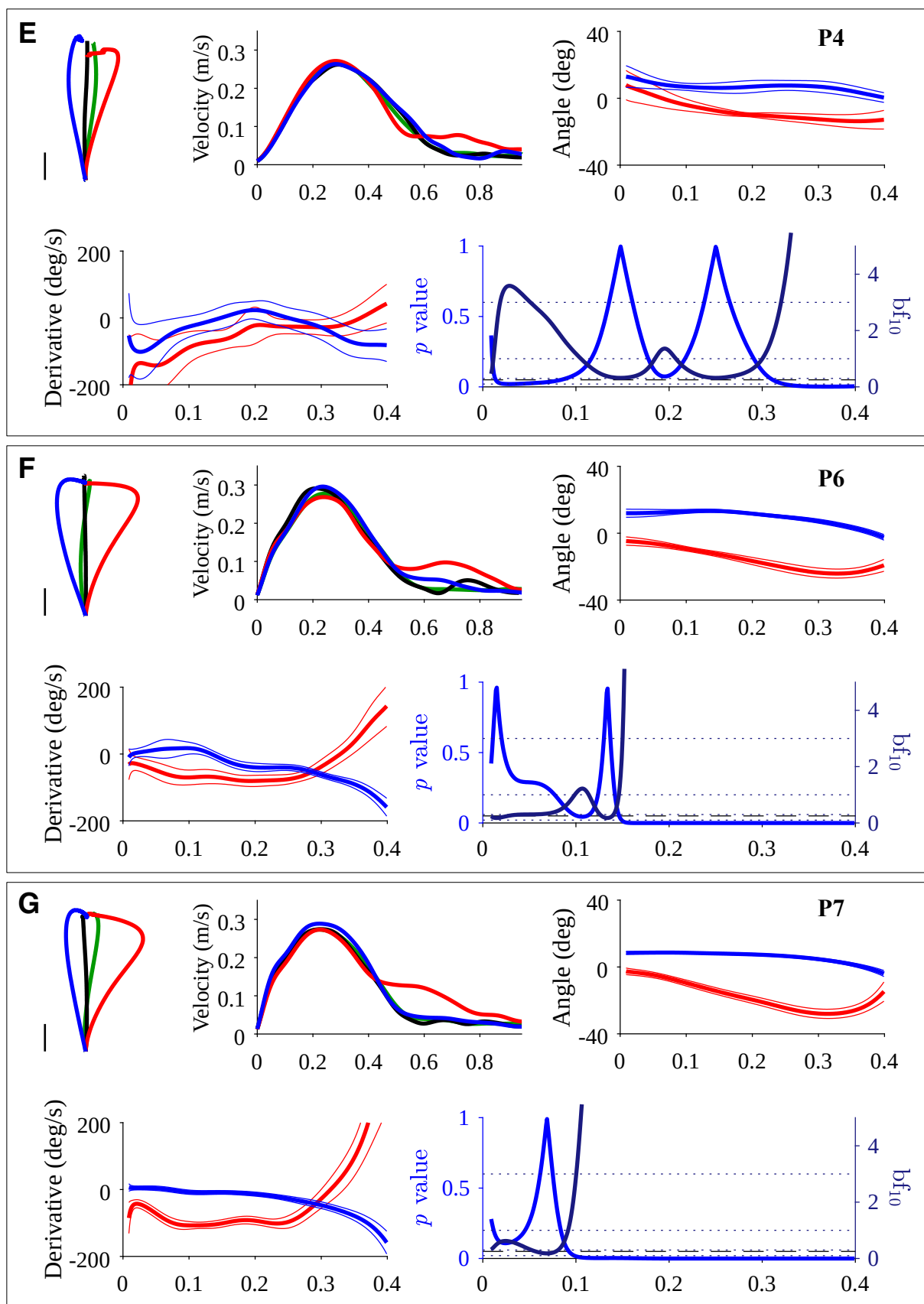

Figure S3

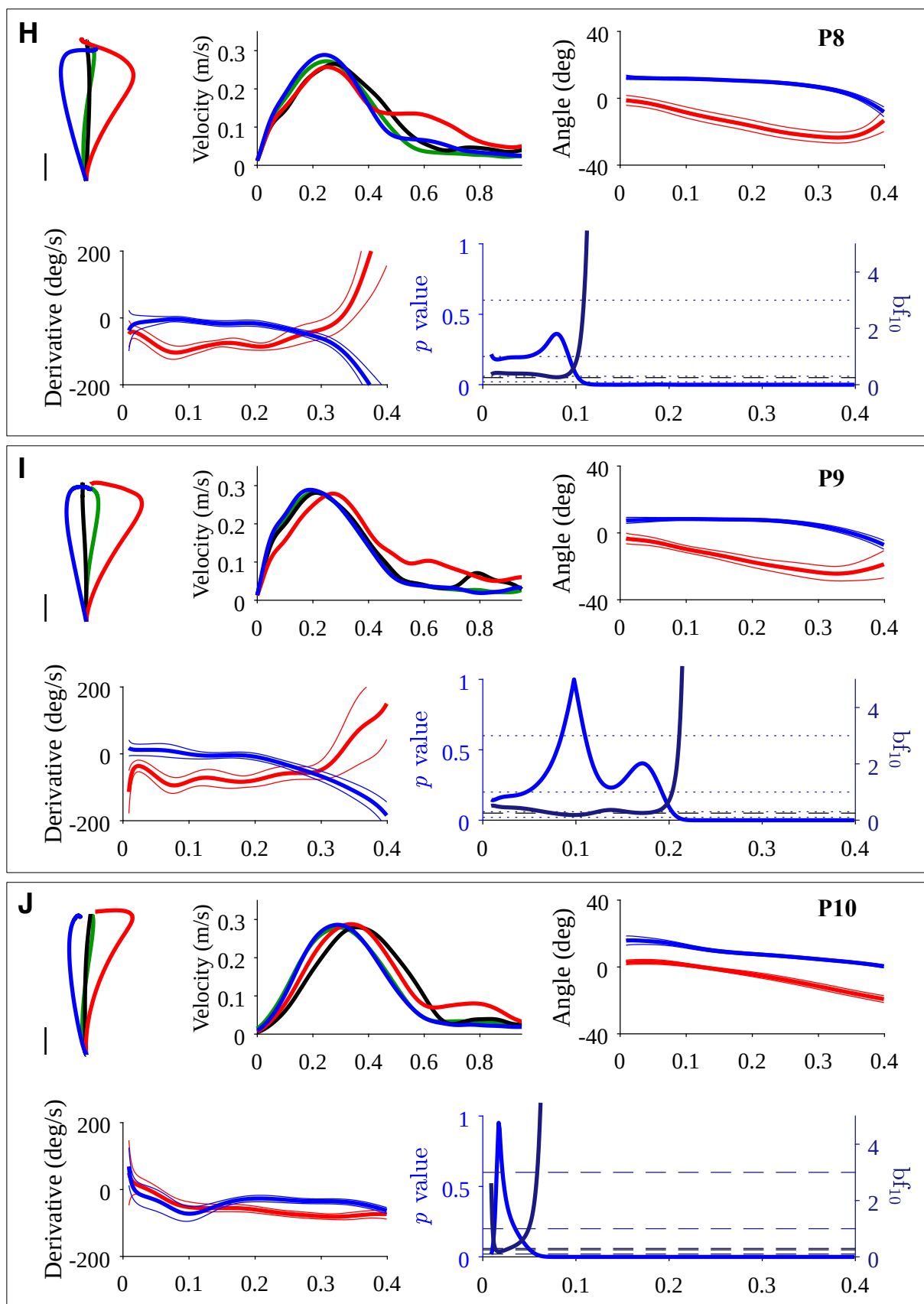

Figure S3

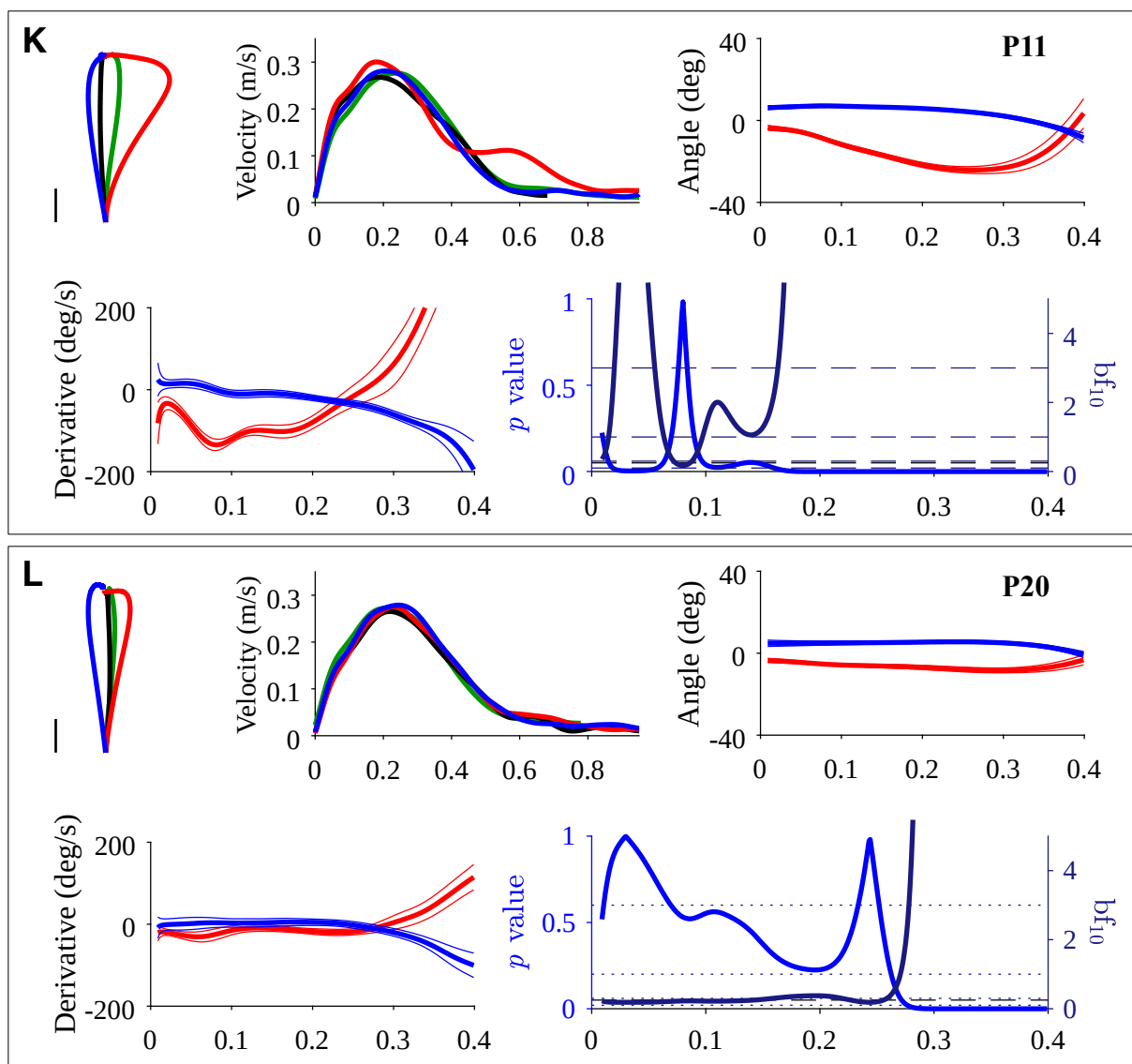

Figure S3

Supplement: S3 Fig — Same format as in Fig 5. For bf10, the dotted lines correspond, from bottom to top, to substantial =, anecdotal =, anecdotal≠, and substantial≠. (PDF) [file pcbi.1010470.s003.pdf]

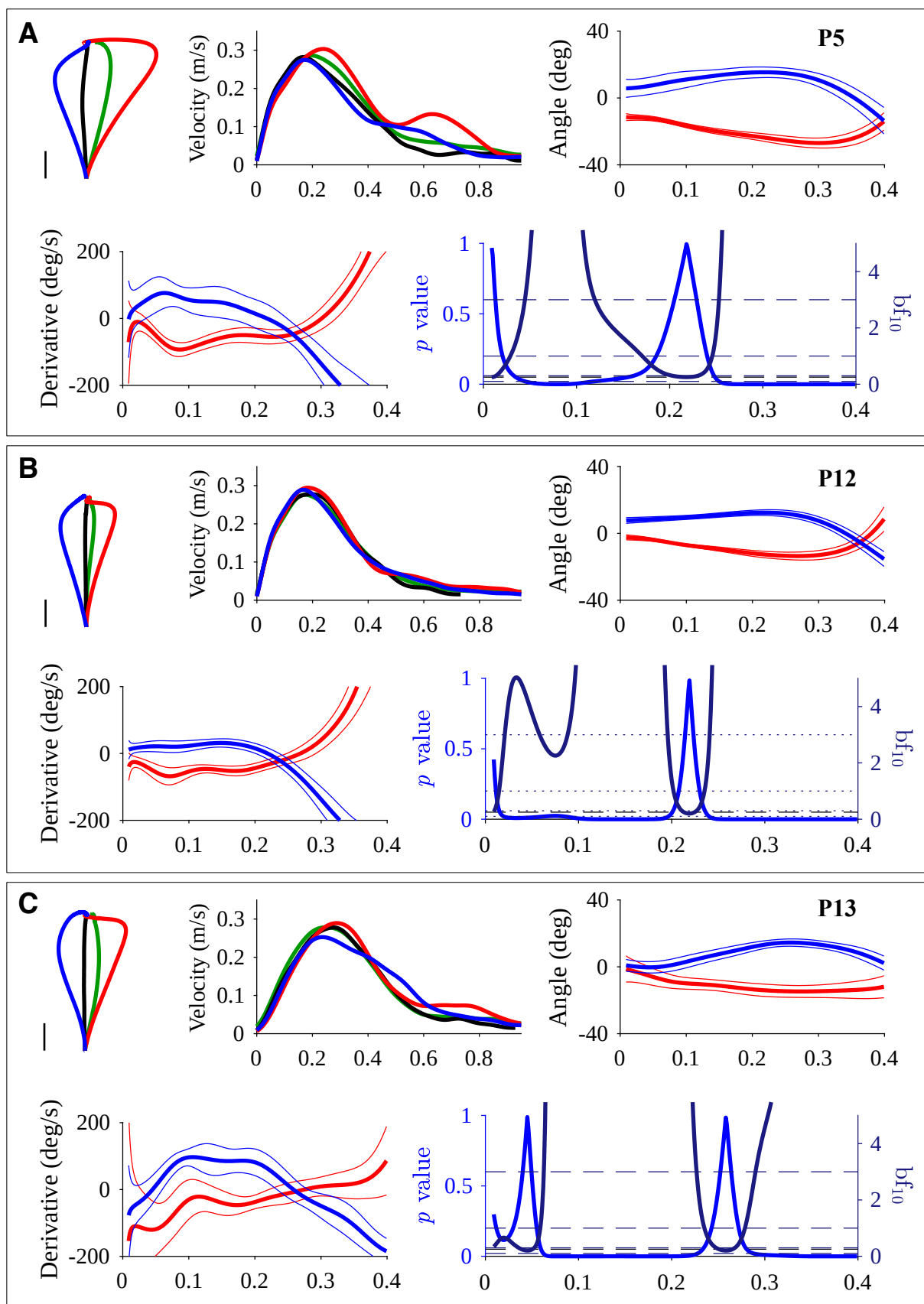

Figure S4

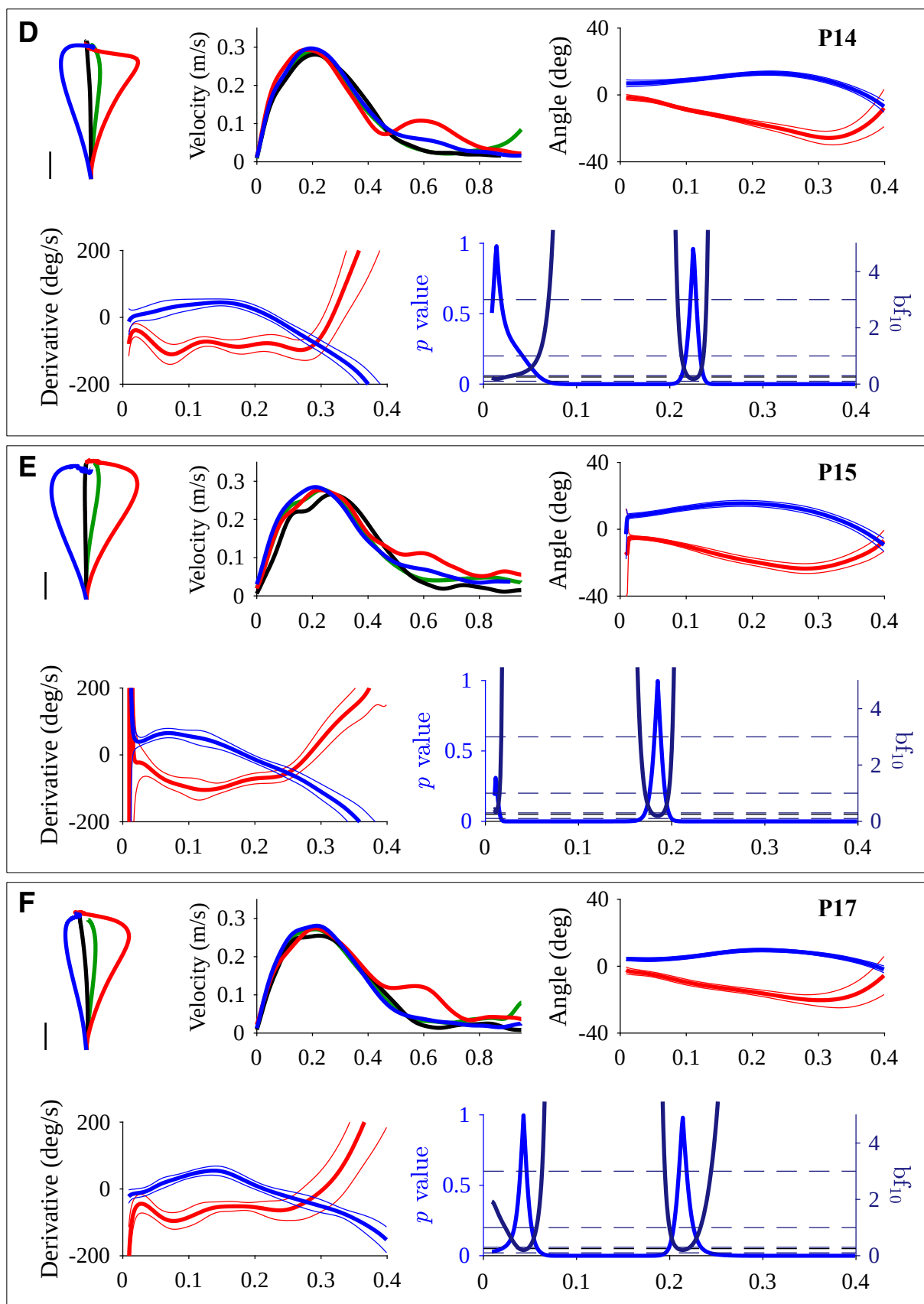

Figure S4

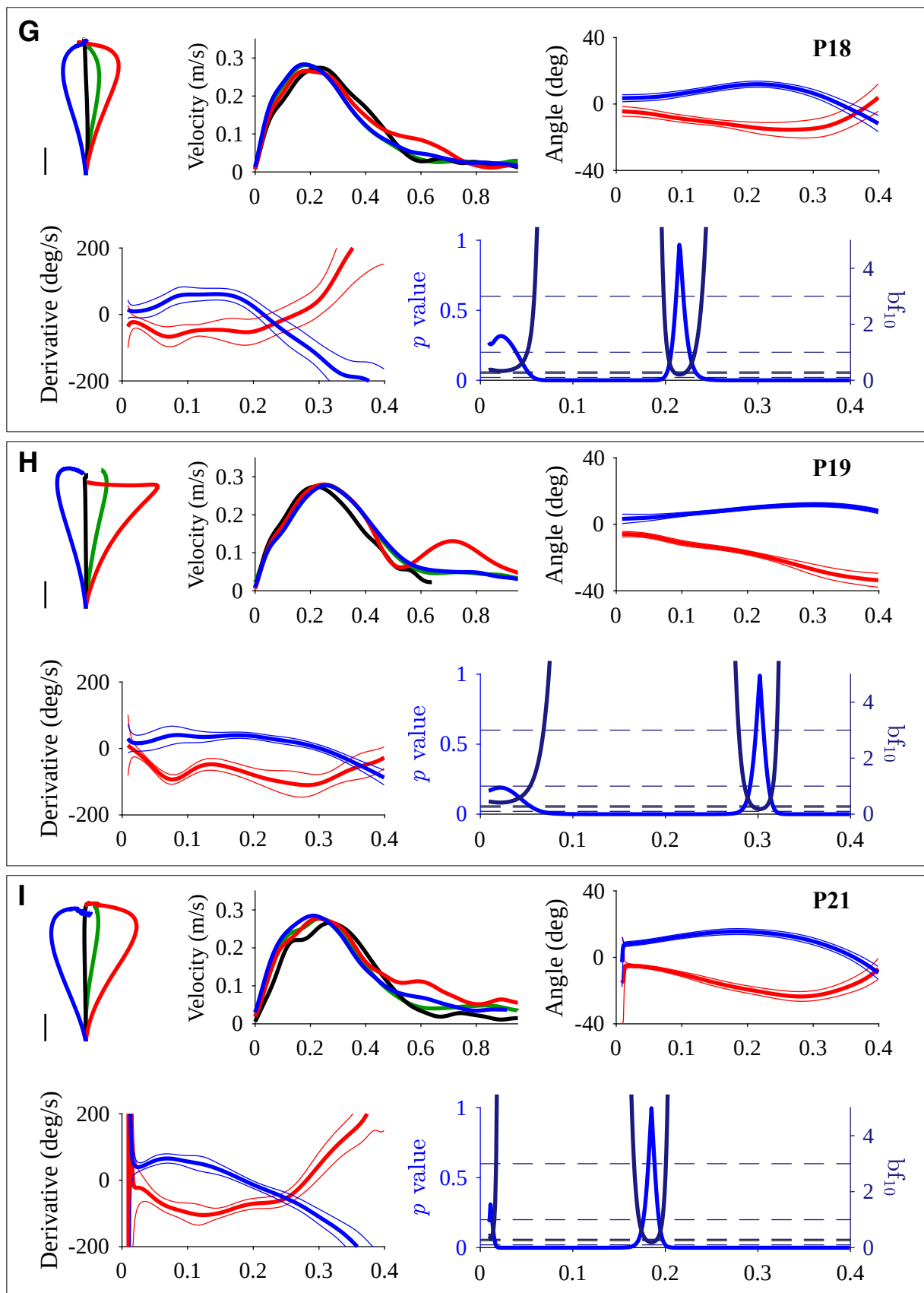

Figure S4

Supplement: S4 Fig — Same format as S3 Fig. (PDF) [file pcbi.1010470.s004.pdf]

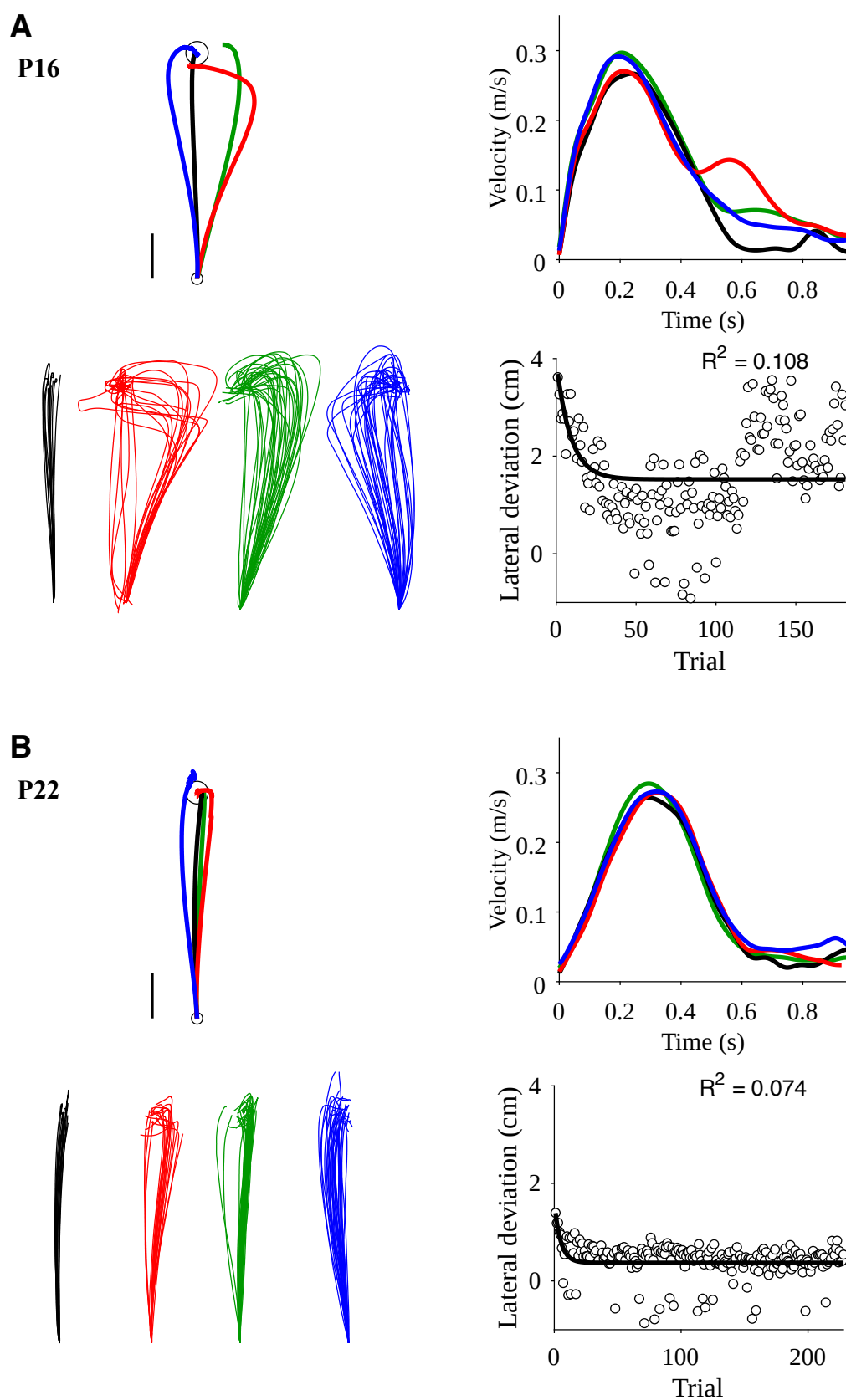

Figure S5

Supplement: S5 Fig — Same format as Fig 5. (PDF) [file pcbi.1010470.s005.pdf]

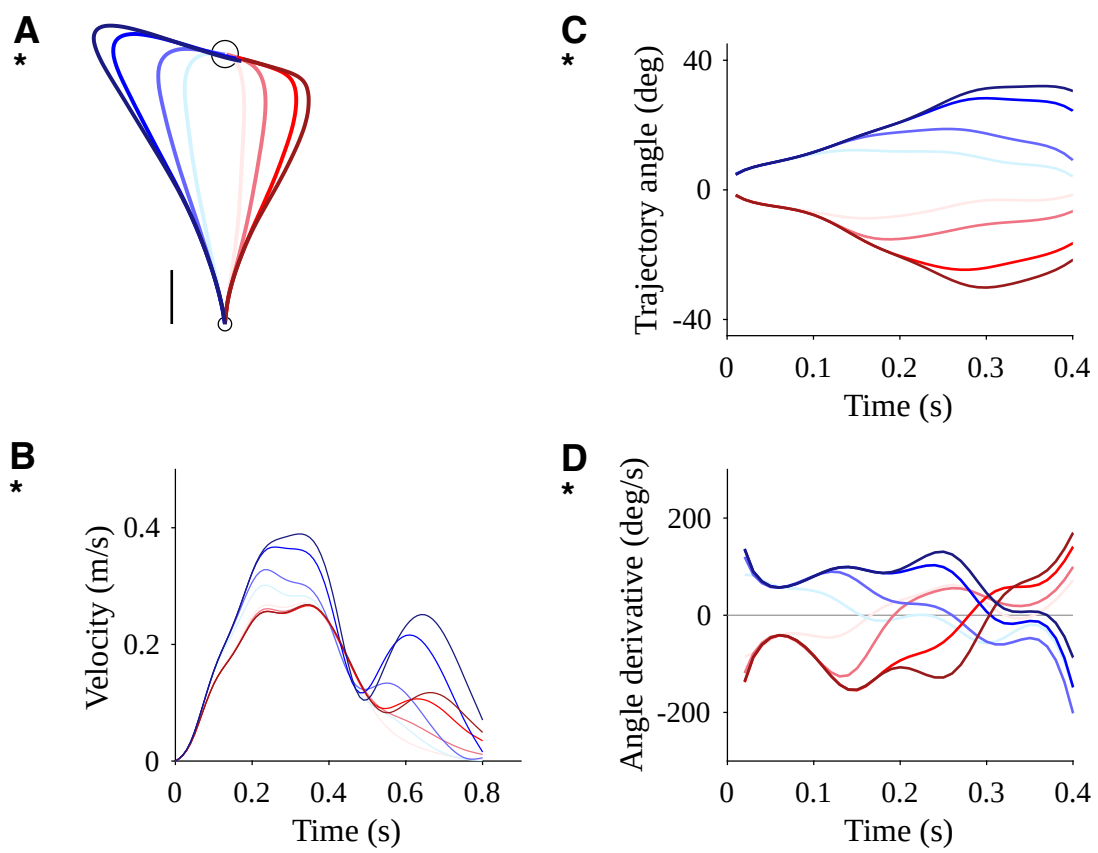

Figure S6

Supplement: S6 Fig — A. Before-effect (red) and after-effect (blue) trajectories. Feedback delay: 0, 0.05, 0.12, 0.15 s; light to dark color. B. Velocity profile. C. Trajectory angle. D. Angle derivative. (PDF) [file pcbi.1010470.s006.pdf]

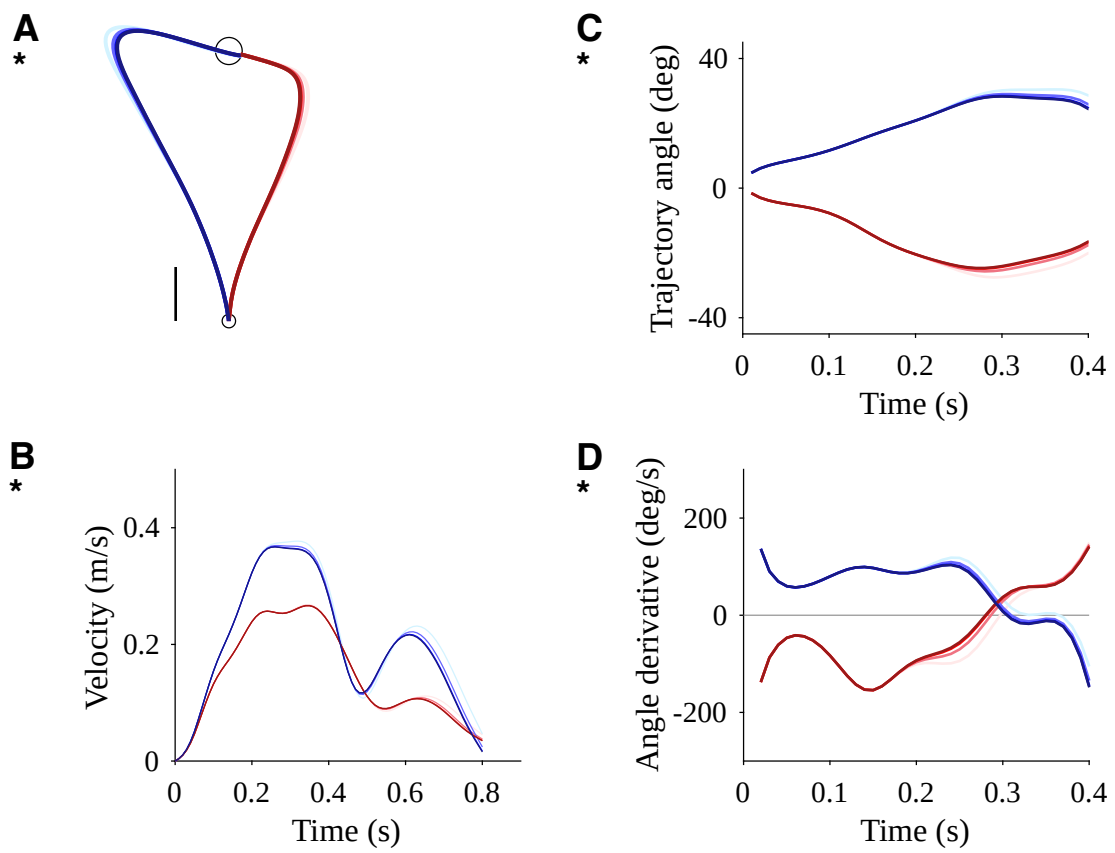

Figure S7

Supplement: S7 Fig — Same format as S6 Fig. Noise ratio σξ/σω (motor/sensory): 0.1, 1, 10, 100; light to dark color. (PDF) [file pcbi.1010470.s007.pdf]

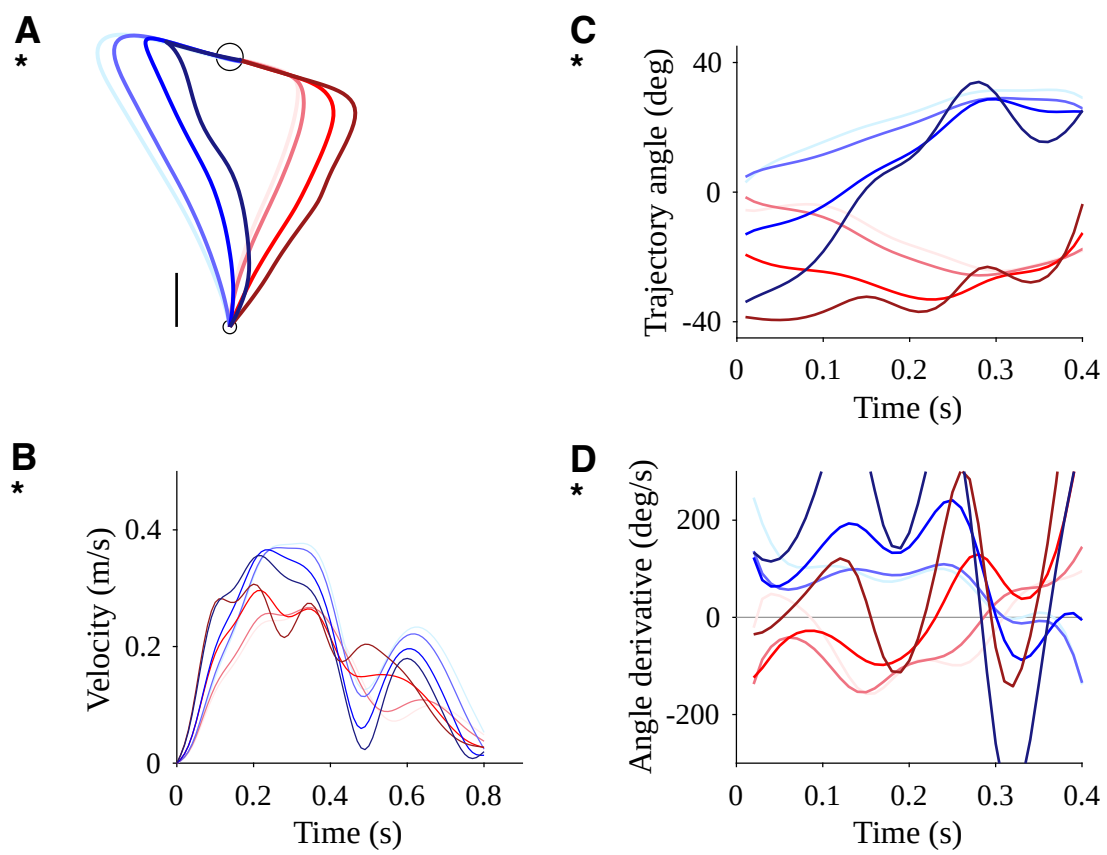

Figure S8

Supplement: S8 Fig — Same format as S6 Fig. Muscle gain ratio gsh/gel (shoulder/elbow): 1, 2, 5, 10; light to dark color. (PDF) [file pcbi.1010470.s008.pdf]

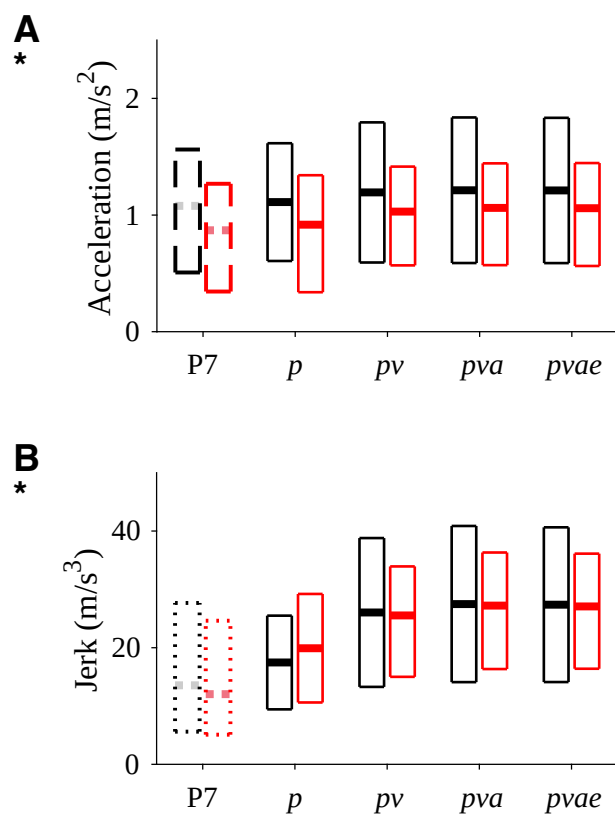

Figure S9

Supplement: S9 Fig — A. Mean and 25–75 percentiles of positive acceleration peaks for baseline (black) and before-effect (red) trajectories for different boundary conditions at via-points: p: only position; pv: position and velocity; pva: position, velocity and activation; pvae: position, velocity, activation and excitation. B. Same as A for jerk. (PDF) [file pcbi.1010470.s009.pdf]

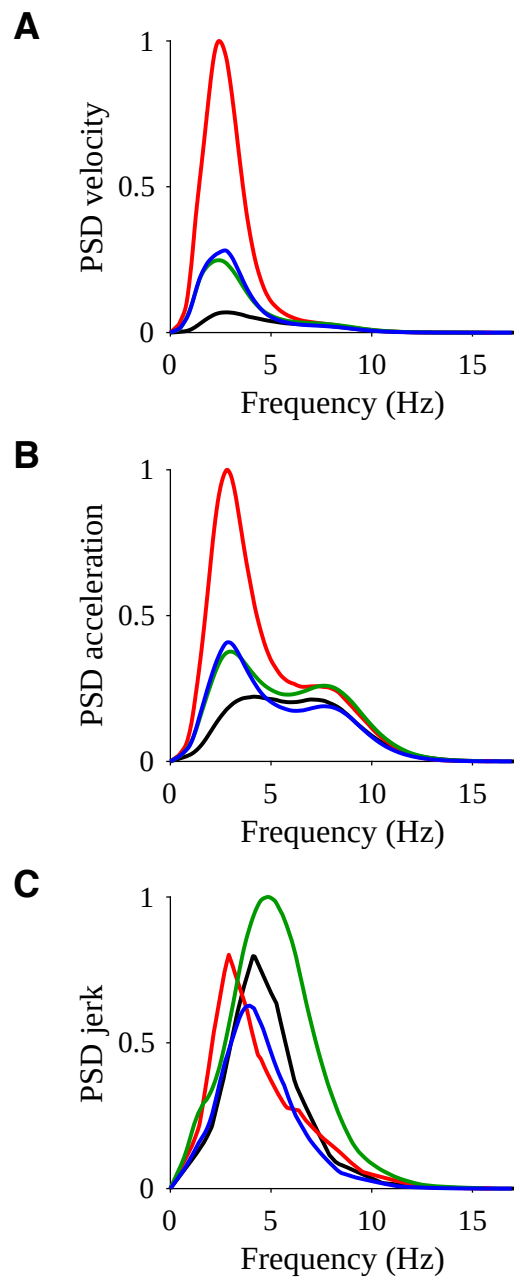

Figure S10

Supplement: S10 Fig — A. Power spectrum density (arbitrary unit) of velocity average across trials and participants, for baseline (black), before-effect (red), adapted (green) and after-effect (green) trials. B. Same as A for acceleration. C. Same as A for jerk. (PDF) [file pcbi.1010470.s010.pdf]
