# Supplementary material for: What is the nature of motor adaptation to dynamic perturbations?
Source: PLoS Comput Biol. 2022 Aug 30;18(8):e1010470. doi: 10.1371/journal.pcbi.1010470 (PMC9467354; doi:10.1371/journal.pcbi.1010470)
Supplement: Attachment — Submitted filename: revPLoS.pdf [file pcbi.1010470.s011.pdf]

Review: **PCOMPBIOL-D-22-00735**
Authors: **Etienne Moullet, Agnès Roby-Brami, Emmanuel Guigon**
Title**: What is the nature of motor adaptation to dynamic perturbations?**

Answer to reviewers' comments

Dear Dr. Gershman,

We would like to thank you for your time and the effort spent to review our manuscript. We are also grateful to the reviewers for their comments and their help to improve our work. You will find below our answers (in **bold**) to the concerns raised by the reviewers (in *italic*), and a description of our corrections to the manuscript (in arial). Modifications are shown in red in the new version of the manuscript.

Sincerly yours,
Etienne Moullet, Agnès Roby-Brami, Emmanuel Guigon

Summary of changes

- We have added a figure (new Figure 1) to better explain the notions of goal selection and action selection. We have modified the **Introduction** accordingly.
- We have added several points to the **Discussion**, in particular regarding (1) the issue of feedforward vs feedback corrections; (2) the respective status of the reoptimization model and the redirection model; (3) the identification of via-points in relation to the redirection model.
- The revised manuscript was reviewed by native English speaker.

Reviewer #1

R#1 *— The authors describe a study aimed at understanding the nature of force field adaptation in reaching movements in humans. They combined empirical studies of FF reaching adaptation with an unusual data analysis technique to distinguish between a model of adaptation that involves learning novel patterns of forces, from a model that instead involves remapping the location of the endpoint target.*

I find the central premise/rationale of the study to be highly problematic. The authors try to draw a distinction between FF learning involving learning a new mapping between states and compensatory forces, and a second view that involves modifying the mapping between intended and actual goals 'irrespective of how to achieve these goals' and they use visuomotor rotation as an example of the latter. (Introduction, second paragraph). It's important to note that in visuomotor adaptation, if participants learn to aim to a different target location, that new actual movement direction does in fact involve new patterns of forces as well. Thus I don't see how one can in principle distinguish between these two models, at least not without some empirical method of disentangling the movement goal from the underlying forces.

A — We used the distinction between goal selection (defined as the "choice of object or location that will require a movement") and action selection (defined as "linking goal and stimuli to a particular movement") as proposed by Krakauer et al. (2019) in their fig. 1. Consider a simplified vectorial description of motor control: a target defines a planned vectorial displacement $\vec{v}_{planned}$ which is converted into an actual vectorial displacement $\vec{v}_{actual}$. Motor control is described by the transformation $\vec{v}_{actual} = AS(GS(\vec{v}_{planned}))$ where GS is the goal selection process and AS the action selection process. Suppose that, under baseline condition, GS and AS are the identity transformation Id, i.e $\vec{v}_{actual} = \vec{v}_{planned}$. Now, we want for some reason (e.g. because a rotation of the visual display is present) that for a given $\vec{v}_{planned}$, $\vec{v}_{actual} = rot_\theta(\vec{v}_{planned})$, where rot_θ is the rotation of angle θ. There are at least two ways to obtain this behavior: $\vec{v}_{actual} = Id(rot_\theta(\vec{v}_{planned}))$ or $\vec{v}_{actual} = rot_\theta(Id(\vec{v}_{planned}))$. In the first solution, the planned vector is rotated ($GS = rot_\theta$) and is entered into an unchanged action selection process ($AS = Id$). There is actually a new motor pattern between $\vec{v}_{planned}$ and $\vec{v}_{actual}$, but not between $rot_\theta(\vec{v}_{planned})$ and $\vec{v}_{actual}$. In the second solution, the planned vector is unchanged ($GS = Id$) and is entered in a modified action selection process ($AS = rot_\theta$). We agree that it is in general difficult to distinguish between these two solutions. Yet this dichotomy is interesting in the framework of adaptation to force fields. The models of Shadmehr and Mussa-Ivaldi (1994) and Izawa et al. (2008) assume that adaptation occurs at the action selection level, i.e. after adaptation, reaching to a target is described as producing a new motor pattern (modified AS) for an unchanged goal ($GS = Id$). These models make specific predictions which are shown in this article: the after-effect trajectories are in mirror with before-effect trajectories. The rationale of this study is the following: if we do not observe a mirror effect in experimental data on force field adaptation, we can conclude against adaptation at the action selection level. See also answer to reviewer #2.

⇒ We have changed the Introduction and added a figure to clarify the notions of goal selection and action selection (new Figure 1).

***R#1** — The authors state (Introduction) that they have not found any study that quantitatively documents the shape of the after-effect trajectories. I think Bhushan & Shadmehr (1999) have done this, no?*

- Bhushan N (1998) A Computational Approach to Human Adaptive Motor Control Shadmehr R, ed. Available at: http://citeseerx.ist.psu.edu/viewdoc/download?doi=10.1.1.392.2697&rep=rep1&type=pdf
- Bhushan N, Shadmehr R (1999) Computational nature of human adaptive control during learning of reaching movements in force fields. Biol Cybern 81:39–60 Available at: http://dx.doi.org/10.1007/s004220050543.

A — There are many articles in which figures illustrate after-effect trajectories. Yet we have not found any study that directly compares the shape of before-effect and after-effect trajectories (e.g., their curvature). This comparison is a fundamental issue with respect to the seminal study of Shadmehr and Mussa-Ivaldi (1994). Their model (compensation) makes specific predictions on the shape of before-effect and after-effect trajectories (Fig. S1).

***R#1** — The authors state (Introduction) that after-effect trajectories seem to resemble "kinematic" trajectories rather than "dynamic" trajectories. I have no idea what this means, which is problematic since this is part of the stated premise/rationale of the study.*

A — The terms kinematic and dynamic are between quotes. As written in the text, they indicate trajectories under kinematic and dynamic perturbations, respectively. An example is shown in the figure below (fig. 2 from Diedrichsen et al. 2005): in the left column (A), from top to bottom, a trajectory from a target jump experiment, a trajectory from a visual rotation experiment (before adaptation), a trajectory from a force field experiment (before adaptation). The main qualitative observations are: (1) for the kinematic perturbations (target jump, visual rotation), the curvature of the trajectory appears to have constant sign; (2) for the dynamic perturbation (curl field), the sign of the curvature changes during the movement.

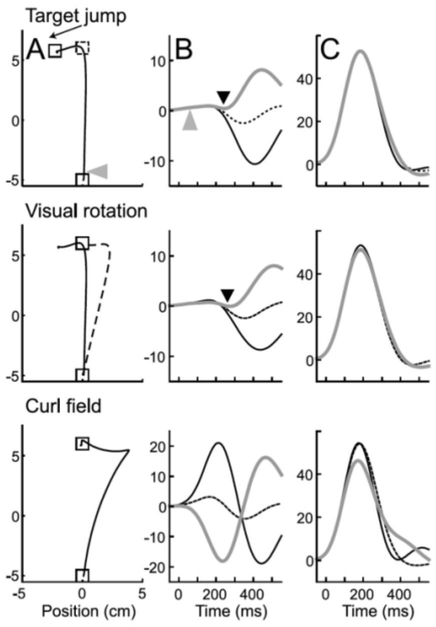

Figure 2 from Diedrichsen et al. (2005), J Neurosci 25:9919.

R#1 *- The authors use a rather unusual method of frequency analysis of kinematics (velocity, acceleration and jerk) in order to distinguish between the competing models described above. I think this is highly problematic, namely using kinematics alone to try to distinguish between models that learn to reach to targets in a force field. It would seem to me the only way to test different models of FF adaptation would be to use models that actually produce forces, e.g. a dynamic model of a two-link planar arm, with for example a hill-type muscle model, and put that model in a simulated FF, and test the two models of control described in the Introduction.*

A — The model is not a kinematic model. As indicated in the first paragraph of Materials and methods, we simulated the displacement of a planar two-link arm using Eq. 1 (arm dynamics) in a null or velocity-dependent force field. The kinematic analysis (velocity, acceleration, jerk in Fig. 3 in the revised manuscript) is used to build precise predictions for the reoptimization model, i.e. to ensure that actual and simulated trajectories have a similar kinematic content.

R#1 *— In fact, using an explicit computational model as described above would enable the reader to fully understand what exactly these competing models are, because in such a computational model one would have to make them perfectly explicit in order to actually run*

the models. This would be my major suggestion for the authors—use a mechanistic model of arm movement that actually generates forces to test competing models of control/learning.

A — This is exactly what has been done.

Reviewer #2

R#2 — *Moullet and colleagues asked, "what is the nature of motor adaptation to dynamic perturbations". They took a fine-grain approach to analyze after-effect trajectories following force-field perturbations and noticed that the data are not consistent with the predictions of a compensatory mechanism in which a mapping was learned between the goal and the optimal forces to achieve the goals in the presence of the applied forces (e.g., after-effects are not a mirror reflection of before-effects). Instead, the after-effects are consistent with a redirection process where participants (implicitly) aim towards a series of spatially remapped targets (i.e., via-points). The fine-grain analysis of after-effect trajectories is rigorous, but the current manuscript seems to miss several important components (see below):*

First, the current version of the manuscript is difficult to follow, and the theoretical differences/underpinnings of the models can be clearer. E.g., which level of behavior do these models try to explain with regards to feedback and feedforward corrections? Some work has suggested that feedforward corrections during late adaptation/aftereffects is a time-shifted version of the feedback corrections during before-effects (Albert & Shadmehr, 2016), whereas others have challenged this idea, positing that feedforward and feedback appear to be more independent (https://drive.google.com/file/d/1Nlhl59X1U8ELzKLTicW1VQSNSCYIRcHd/view). The current manuscript does not address this critical issue.

A — Our study is not concerned with how participants adapt trial-by-trial to the force field. We only model the adapted state as being either a consequence of a reoptimization process or a redirection process. We have no specific information to add in the debate between feedforward and feedback corrections.

⇒ We have added this point in the **Discussion**.

R#2 — *The authors bring up that the models differ in how they alter different stages of movement (e.g., action selection, goal selection) – however, this terminology can be used differently across papers. For example, some refer action selection as where people explicitly aim (Kim et al., 2020; Krakauer et al., 2019), but in this paper, goal selection seems to correspond to where people explicitly aim. Clarifying how these terms are used in this paper would help readers follow the logic/differences among these models (a schematic may help).*

A — The notions of goal selection and action selection can be clarified in the following way (see also answer to reviewer#1). The motor system contains (new Fig. 1A): (1) a process, called goal selection (*GS*), which provides the current goal for a given task; (2) a process, called action selection (*AS*), which translates a current goal (e.g. a target to reach) into the proper displacement of the current effector (e.g. the arm) toward the goal (the target). In a baseline condition (e.g. we see a target and we move our arm toward the target), the two processes are not easy to distinguish. Yet two conditions are interesting to discuss.

First, we consider the case of a rotation of the visual display (angle θ; Fig. 1C, left). A visual target appears and the task objective is to displace the visual feedback of the hand toward the target. One solution is to select a goal which is a transformed target at an angle $-\theta$ with respect to the visual target (GS) and to aim (AS) toward this transformed target (Fig. 1C, right). In this case, the GS is a rotation (by $-\theta$) and AS is unchanged compared to a baseline condition without visuomotor rotation. An alternative solution is to aim toward the original target (unchanged GS) using a modified AS (rotation by $-\theta$; Fig. 1C, center).

Second, we consider the case of a force field perturbation (Fig. 1B, left). A visual target appears and the task objective is to displace the hand toward the target in the presence of a force perturbation. One solution is to aim toward the target (unchanged GS) using a modified AS which includes a compensation for the applied force (Fig. 1B, center). The models of Shadmehr and Mussa-Ivaldi (1994) and Izawa et al. (2008) correspond to this solution. They assume that adaptation occurs at the action selection level, i.e. after adaptation, reaching to a target is described as the production of a new motor pattern for an unchanged goal. An alternative solution is to assume that the AS remains unchanged during adaptation, and adaptation results from aiming toward a series of new goals in such a way that the combined effect of aiming to these goals and the perturbation makes the arm moving straight toward the target (Fig. 1B, right).

⇒ We have changed the Introduction and added a figure to clarify the notions of goal selection and action selection (new Figure 1).

R#2 *— Second, missing model comparisons. The authors have focused on whether different models can qualitatively predict the after-effect trajectories. However, have the authors addressed the differences in model complexity (e.g., number of parameters, or number of assumptions of these models)? The "winning" model (redirection model) includes the notion of via-points, each of which make up the entire trajectory. Do the number of via-points change the model complexity, and therefore, make the re-direction model less parsimonious? An analysis of AIC and BIC may be useful to arbitrate among these models.*

A — The main result of the article is the observation that the reoptimization model cannot account for the characteristics of after-effect trajectories following adaptation to a velocity-dependent force field. Accordingly, we propose to reject this model. On this basis, there is no rationale to compare this rejected model to any other model. The fact that the redirection model is less parsimonious than the reoptimization does not argue in favor of the reoptimization model.

R#2 *— Third, missing tests of alternative hypotheses. The current conclusions are largely based on one observation, the trajectory of the aftereffect data. While the re-direction model does a decent job capturing this behavior (but perhaps not parsimoniously, see Point 2), the data may also be consistent with one or more of the following:*

a) A model of Proprioceptive re-alignment (or PReMo; see Feature 6 of (Tsay et al., 2021)): participants following forcefield perturbation may experience a shift in their sensed hand position towards the direction of the force-field (Ostry et al., 2010). PReMo provides a theoretical account for the feedforward corrections (although feedback corrections are not considered and thought to arise from another learning system).

A — The rationale of our study is to consider computational models which can be simulated and generate quantitative predictions on the shape of trajectories in force fields. PReMo is not formulated as a model which explains trajectory formation in a force field. As it is, it cannot be considered as an alternative hypothesis.

R#2 — *b) The presence of cognitive re-aiming strategies: The authors note in the Discussion that the re-direction model does not arise from a change in explicit re-aiming in response to a force-field. Can the authors rule out this explanation? There have been a study that shows explicit re-aiming in response to forcefields (Schween et al., 2020). Aftereffects may also arise from explicit re-aiming if instructions are ambiguous (e.g., "reach directly to the target" could either refer to brining the hand or the invisible cursor to the target; if referring to the invisible cursor, participants may still explicitly re-aim away from the original target during the washout aftereffect period).*

A — We tried to be very cautious on the contribution of implicit and explicit processes to motor adaptation. In fact, our study does not provide any information on this issue. We simply noted that the proposed redirection process should not be considered as an "explicit cognitive strategy" (term taken from Mazzoni and Krakauer 2006) in the sense that redirection is the result of a learning process (i.e. after adaptation to the force field).

R#2 — *c) Before-effects time-shifted to form after-effects: Can the compensatory model be salvaged if a gain (or time shift) parameter was included? That is, can the authors experimentally or logically rule out the notion that after-effects are just a time-shifted version of the before-effects (i.e., people are selecting the same goal, that is, the original target), rather than a re-direction towards another target?*

A — Our approach was the following. We considered an optimal feedback control model and we adjusted some parameters to match experimentally observed baseline and before-effect trajectories (Fig. 3 in the revised version). Once the model was adjusted, reoptimization was applied and the adapted and after-effect trajectories were emergent properties (Fig. 4). At this stage, there was no way to further adjust the after-effect trajectories. In this sense, the notion that after-effects are just a time-shifted version of the before-effects is not directly relevant as modeling is concerned. If the reoptimization model or the redirection model produce after-effects which are a time-shifted version of the before-effects, we would favor an explanation in terms of a model (reoptimization or redirection) rather than in terms of a consequence of the model (time-shifting).

R#2 — *d) while the re-direction is a viable hypothesis, the critical idea (i.e., people are redirecting their movements to spatially re-mapped targets) was not directly tested (currently, only inferred via model comparison). Is there a way to probe where people are aiming, or the location of these via-points directly? If it's explicit, then the authors could consider using an aiming wheel + aim report method. If these via points are implicit, does the re-direction model generate any qualitatively different predictions with regards to how aftereffects generalize to local/global targets, compared to the compensation hypothesis?*

A — Our strategy was first to reject the reoptimization model and then to promote the redirection model as a possible alternative (if adaptation is not at the action selection level, it could be at the goal selection level). The redirection model has not been specifically designed for adaptation. It is a general model of motor control that inherits from the model of Todorov and Jordan (2002) and has many interesting properties (Guigon et al. 2019; Guigon 2022). The via-points are a central element of the model to explain task

representations (Guigon 2022) and the presence of kinematic fluctuations in motor signals (Guigon et al. 2019). We agree that the critical idea of the redirection model was not directly tested. There are two reasons for this. First, it was not in the objectives of this study. The main objective was the test of the reoptimization model. The failure of the reoptimization model paved the way for the promotion of the redirection model. Second, we think that there is little way to find the location of via-points. It is tempting to assume that variability should be minimum at the via-points as in the experiment of Todorov and Jordan (2002) in which the via-points are indicated to the participants (their fig. 3). Unfortunately, the kinematic variability is so large (e.g. Fig. 3G) that we cannot expect anything precise from an analysis of variability, suggesting that in the absence of a constrained trajectory, via-points may be themselves subject to variability from trial to trial.

⇒ We have added this point in the **Discussion**.

Reviewer #3

R#3 *— This study examined whether motor adaptation to a dynamic (force) perturbation reflects changes at the action selection level vs at the goal selection level. The authors performed a strict examination of arm trajectories as subjects made reaching movements with one arm to a visuomotor target. A velocity-dependent force field disrupted the reaching movements. The "change in action selection" model predicts mirror-symmetric shapes for the arm trajectories early in adaptation and the after-effect trajectories. This was not found, however. Instead, the afer-effect trajectories suggest that the process of adaptation occurs instead at the goal selection level. By selecting a new goal, the motor system has the option to generate arbitrary trajectories, which in some cases might be more efficient.*

I have no problems with the manuscript. The topic is important as it sheds light on how much the motor system controls trajectory details--more than was expected. The approach is sound, the results are clear, and the figures are great. The results are interesting and they will help advance our understanding of human gait control.

I have one comment regarding a premise of the study's hypothesis:

p. 4, par. 2. "after-effect trajectories, ... should incorporate a negative image of the forces induced by the applied force field..." A 2015 study by Krakauer's group showed that the amount of after-effect in rotation learning can be influenced, and even entirely cancelled, by the number of trials performed at asymptote. The interpretation was that reward-related components of motor adaptation.

A - We are unsure about the meaning of this comment. A possible interpretation is the idea that after-effects are defined not only by the nature of the perturbation but also by the adaptation protocol. This is the case at least for adaptation to a visuomotor rotation (Vaswani et al. 2015).

⇒ We have added this point at the beginning of the **Discussion**.

R#3 *— Minor suggestions:*

Minor changes are needed concerning several instances of awkward word choice or use of prepositions. There are many phrases, throughout the manuscript, that are either grammatically incorrect or that vary from typical English usage. I give a few examples below,

but this list is not complete. It would be best to have the manuscript revised by a reader with fluent mastery of English.

Examples:

p. 2, par. 2: delete "against reality."

p. 2, par. 2: Change "The results suggest to change our mind..." to something like "The results change our view of motor adaptation."

p. 3, par. 1: change "cooperation" with something like "balance"; change "mandatory" to "necessary."; change "outrageously" to "disproportionately."; delete "upon request."

⇒ The revised manuscript was reviewed by native English speaker.
